# A Unique Case of Squamous Cell Carcinoma Presenting Within a Lesion of Granuloma Annulare

**DOI:** 10.7759/cureus.27845

**Published:** 2022-08-10

**Authors:** Morgan L Zabel, Tyler Evans, Adam V Sutton, Matthew Stephany

**Affiliations:** 1 College of Medicine, University of Nebraska Medical Center, Omaha, USA; 2 Dermatology, University of Nebraska Medical Center, Omaha, USA

**Keywords:** general dermatology, dermatopathology, granuloma annulare, cutaneous squamous cell, medical dermatology

## Abstract

Granuloma annulare (GA) is a common benign granulomatous inflammatory disorder of the dermis or subcutis with classic morphologic and histologic presentation. Common clinical subtypes of granuloma annulare include localized, generalized, subcutaneous, perforating, and patch types. A biopsy is critical in cases of granuloma annulare with atypical features. We present a case of a 58-year-old male who presented with an annular scaly erythematous plaque on the right dorsal hand. Shave biopsy demonstrated irregular nests of mildly atypical squamous epithelium present within the superficial dermis, with abundant histiocytes and multinucleated giant cells arranged in palisades peripherally. These findings were consistent with invasive well-differentiated squamous cell carcinoma (SCC) with surrounding granuloma annulare. This case highlights a unique presentation in which clinicopathologic correlation is critical prior to arriving at a correct diagnosis.

## Introduction

Granuloma annulare (GA) is a common benign granulomatous inflammatory disorder of the dermis or subcutis with classic morphologic and histologic presentation. We present a case where clinicopathologic correlation led to the diagnosis of invasive squamous cell carcinoma (SCC) within a well-established lesion of GA. To our knowledge, this is the first report of these two diagnoses coexisting as a single lesion in the literature. Morphologic presentation of GA is commonly annular or arcuate, non-scaly, red to brown papules or plaques hypopigmented centrally relative to the periphery. While there are several variants of GA, the localized variant is the most common subtype. The hands, feet, arms, and legs are the sites of predilection in approximately 80% of cases [1]. Histologically, GA presents with a focus on necrobiosis surrounded by palisading histiocytes, with mucin deposition being a hallmark feature [2].

Despite GA’s classic presentation, it has been known to mimic other diagnoses. Further, other conditions have been reported to masquerade as GA, including borreliosis, Kaposi sarcoma, and tuberculoid leprosy [3]. Our case, in addition to these reports, further demonstrates the importance of clinicopathologic correlation in working up cases of suspected GA.

## Case presentation

**Figure 1:**
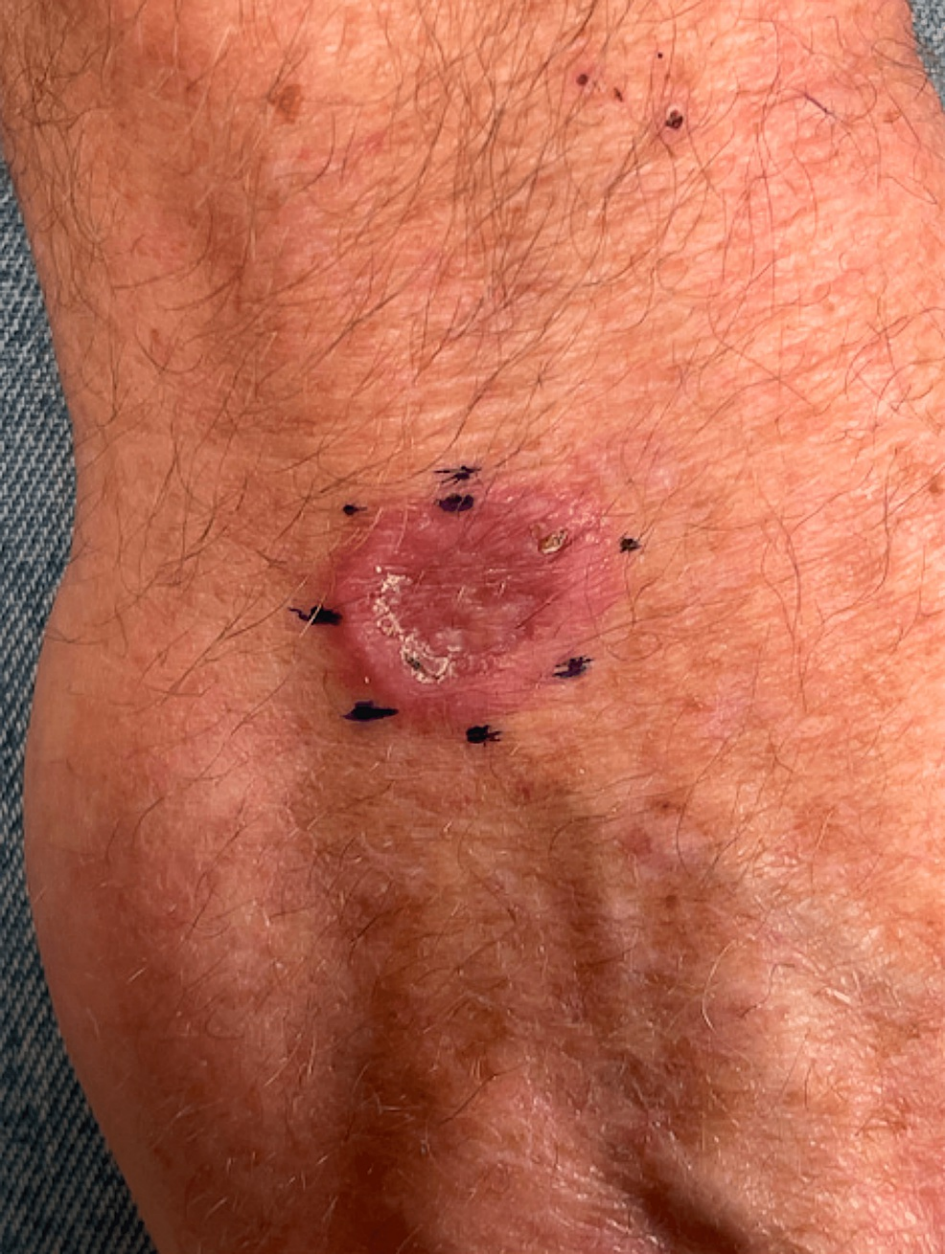
Clinical examination of the right dorsal hand Erythematous plaque measuring 2.1 × 1.6 cm with rolled borders, central scale, and dilated vasculature

**Figure 2:**
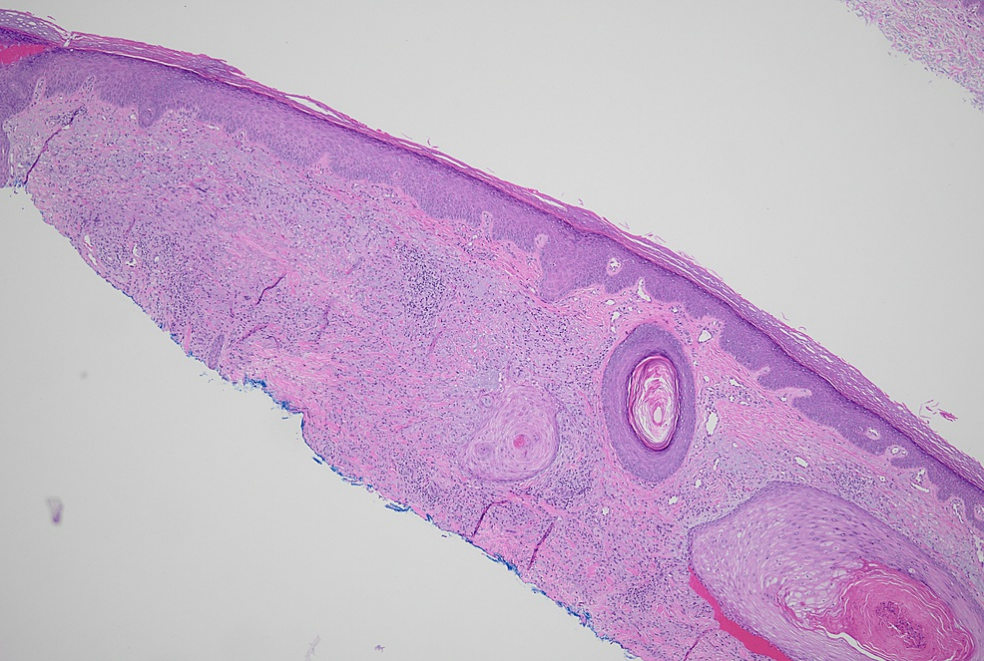
Histopathologic examination of a shave biopsy from the right dorsal hand Irregular nests of mildly atypical squamous epithelium present within the superficial dermis centrally, with surrounding granulomatous dermatitis characterized by histiocytes and multinucleated giant cells arranged in palisades (H&E, 40×)

A 58-year-old male presented with a new raised skin lesion on the right dorsal hand. He reported that the lesion had grown for a couple of months. He denied any trauma to the area. He denied a history of similar lesions. He denied any systemic symptoms, including fevers, chills, weight loss, and night sweats. He denied a prior personal or family history of skin cancer. On examination, there was a 2.1 × 1.6 cm annular pink plaque with raised borders and central erosion located on the right dorsal hand (Figure 1). Morphology was consistent with granuloma annulare (GA); however, secondary changes raised concern for non-melanoma skin cancers. Histopathologic examination demonstrated irregular nests of mildly atypical squamous epithelium present within the superficial dermis centrally, with surrounding granulomatous dermatitis characterized by histiocytes and multinucleated giant cells arranged in palisades (Figure 2). There was no significant presence of elastophagocytosis, ruling out actinic granuloma.

These findings were consistent with invasive well-differentiated squamous cell carcinoma (SCC) with surrounding GA, and the patient was referred for Mohs micrographic surgery (MMS). The patient was cleared by MMS after 1 stage. The preoperative size was 27 × 25 mm, and the postoperative defect was 32 × 35 mm. The defect was partially closed using the purse-string closure technique with the center left open to secondary intent.

## Discussion

GA is a granulomatous inflammatory disorder thought to be most common among young adults, especially women [4]. While the lesion itself is benign, GA is thought to possibly be associated with many conditions inducing thyroid disease, diabetes mellitus, infection, and malignancy [5]. Subtypes of GA include localized, generalized, subcutaneous, perforating, and patch types. Localized GA is the most common and is likely to resolve spontaneously, whereas generalized GA is rare and may persist for decades [6]. Treatment depends on the clinical subtype and the extent of involvement. Localized variants typically do not require treatment intervention and resolve within two years [7]. Topical corticosteroids can be helpful for pruritic and symptomatic lesions. Due to its chronic and disseminated nature, generalized GA can be treated systemically. Several reported therapies include retinoids, dapsone, hydroxychloroquine, cyclosporine, and TNF-alpha inhibitor, although there are very few clinical trials validating those therapies [8].

Due to its classic morphologic presentation, GA is often a clinical diagnosis. The absence of scale and other secondary features can help assist in the diagnosis of GA [9]. However, the presence of additional features in a lesion of suspected GA such as increased vasculature or erosion should prompt further investigation and workup to exclude other diagnoses, including malignancy. We report a case where secondary surface change led to a biopsy and the diagnosis of invasive SCC within a classic lesion of localized GA. These diagnoses may not have been previously reported as co-occurring due to the relatively high incidence of GA.

SCC has been known to arise in chronic wounds, known as a Marjolin ulcer [10]. The origin of SCC in this case is unclear as there is a lack of representation of these two diagnoses co-occurring in a “single” lesion in the literature. Additionally, granulomatous inflammation is a known reaction to occur with SCC, and the hallmark histopathologic features of GA may have previously gone unnoticed [11]. With the assistance of clinical correlation, GA can be distinguished from granulomatous inflammation on histopathology by containing focal areas of necrobiosis surrounded by palisades of histiocytes in the dermis with characteristic mucin deposition [12]. Additionally, GA can be distinguished from actinic granuloma by lack of significant elastophagocytosis.

## Conclusions

GA is a benign granulomatous lesion that is typically a clinical diagnosis. The absence of surface change and scale is a crucial finding in differentiating GA from other malignant lesions. A biopsy is critical in cases of GA where morphology is atypical. This case demonstrates a rare presentation of both SCC and GA presenting in a “single” appearing lesion. Importantly, this case highlights a unique presentation for which clinical correlation is imperative prior to arriving at a correct diagnosis and treatment intervention.
